# Rapid diffuse pleural thickening due to metastatic meningioma

**DOI:** 10.1002/rcr2.313

**Published:** 2018-02-28

**Authors:** Fumi Kobayashi, Takeshi Saraya, Kosuke Ohkuma, Masachika Fujiwara, Hajime Takizawa

**Affiliations:** ^1^ Department of Respiratory Medicine Kyorin University School of Medicine Mitaka City Japan; ^2^ Department of Pathology Kyorin University School of Medicine Mitaka City Japan

**Keywords:** Anaplastic meningioma, malignant mesothelioma, metastasis, pleural thickening

## Abstract

A 64‐year‐old man was referred to our hospital because of persistent dyspnoea for the past 1 month. He had recurrent brain anaplastic meningioma after two operations and irradiation. He suffered from right pleural effusion in the previous few months and was diagnosed with malignant mesothelioma via pleural biopsy 1 month prior to coming to our hospital. At his first visit to our hospital, thoracic computed tomography demonstrated rapidly developed large inhomogeneously enhancing pleural thickening up to 3 cm, which surrounded the right hemithorax, together with left‐sided pleural effusion. After re‐evaluation of the pathological specimens retrieved from the local hospital, he was finally diagnosed with pleural metastasis secondary to anaplastic meningioma (WHO classification, grade 3). Generally, brain meningiomas are believed to be benign and seldom metastasize to other organs. However, the present case clearly demonstrated the unique clinical presentation of anaplastic meningioma, also known as malignant meningioma, which mimicked the pathological and radiological findings of a malignant mesothelioma.

## Introduction

Extracranial metastases from brain meningioma have a low incidence of 0.76% 1 and occur mostly in the lungs, bone, intraspinal, abdominal viscera, mediastinal, and cervical lymph nodes. However, pleural metastasis can occur as an extremely rare complication of brain meningioma regardless of the tumour grading 2. Here, we report a case with metastatic meningioma to the pleura that radiologically and pathologically mimicked malignant mesothelioma.

## Case Report

A 64‐year‐old man was referred to our hospital because of recent dyspnoea of a duration of 1 month. One month prior to his referral, he was diagnosed with biphasic malignant mesothelioma at his local hospital based on pathological findings of the pleura after video‐assisted thoracic surgery. He was an ex‐smoker with a history of 42 pack‐years and had denied any exposure to dust. Two years before referral, a resection of an identified meningioma located at the right parasagittal sinus was performed, and he was diagnosed with anaplastic meningioma, (World Health Organization (WHO) classification, grade 3) 3. However, the tumour in the frontal convexity recurred 1 year later; therefore, a second operation and brain irradiation were performed. At his last evaluation, 1 month before presenting to our hospital, enhanced magnetic resonance imaging of the brain showed a residual tumour measuring 1.6 cm in size (Figure not shown).

**Figure 1:**
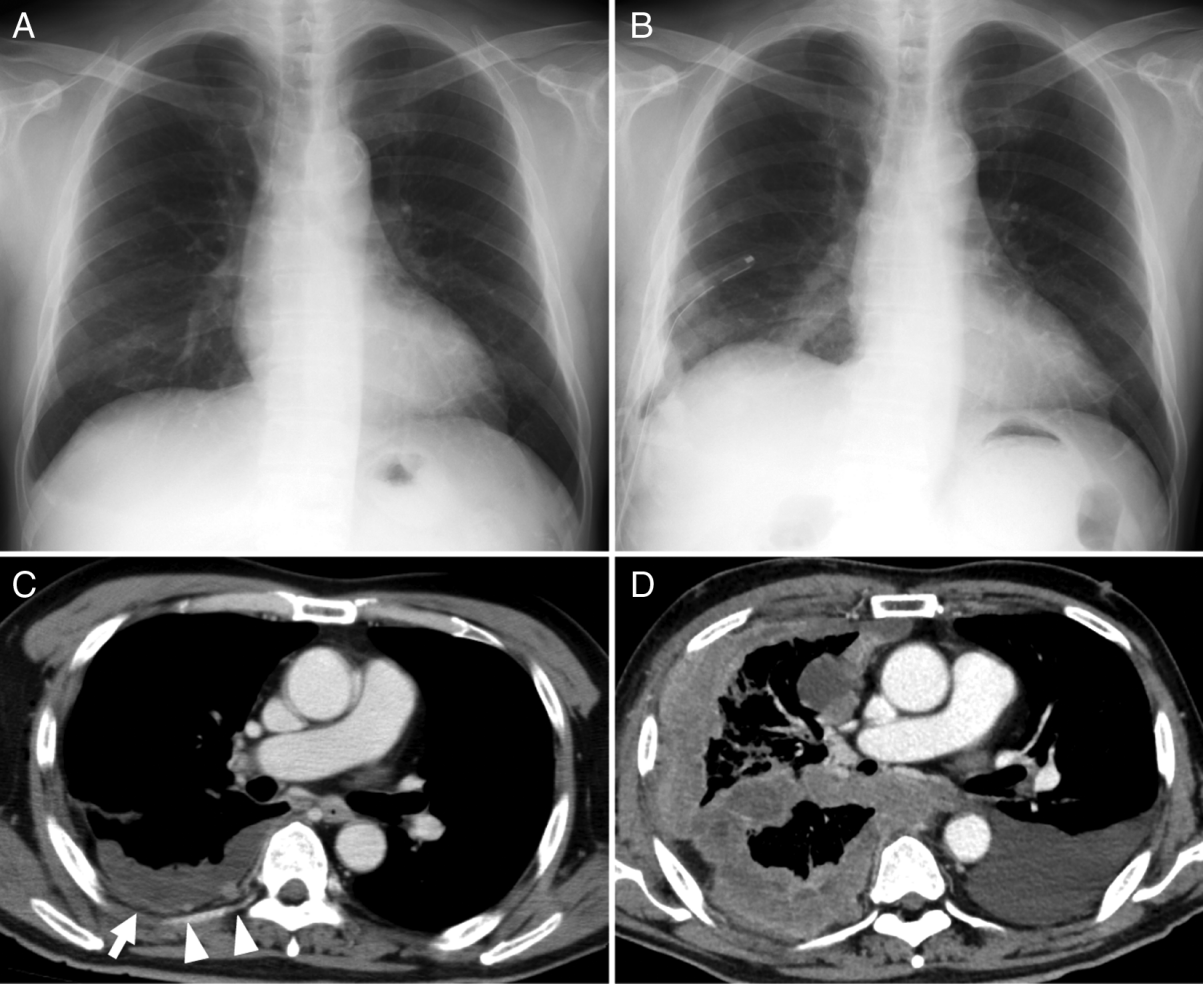
(A) Normal chest X‐ray performed 4 months prior to referral. (B) Chest X‐ray performed 1 month prior to referral, after the insertion of thoracic drainage, showing an infiltration of the right lower lung field with blunting of the costophrenic angle. (C) Thoracic CT performed 1 month prior to referral. Arrowhead depicts the presence of small amounts of right pleural effusion with enhancing parietal pleural thickening or tiny nodules. (D) Thoracic CT on referral depicts the emergence of a large inhomogeneously enhancing pleural thickening measuring up to 3.4 cm and extending to the entire right hemithorax, together with left‐sided pleural effusion. CT, computed tomography.

A chest X‐ray performed 4 months earlier (Fig. 1A) appeared normal. However, his right pleural effusion progressed in the following 2 months, and thoracic drainage was performed at his local hospital. After removal of the right‐sided pleural fluid 1 month prior to his referral to us, a chest X‐ray demonstrated an infiltration of the right lower lung field with blunting of the costophrenic angle (Fig. 1 B), enhanced parietal pleural thickening (arrow head, Fig. 1C), and pleural effusion on enhanced thoracic computed tomography (CT). Furthermore, a small number of tiny nodules were also noted in the parietal pleura (Fig. 1C, arrow head).

Surprisingly, thoracic CT performed at his first visit to our hospital depicted the emergence of a large inhomogeneously enhancing pleural thickening measuring approximately 3 cm, which surrounded the right hemithorax (Fig. 1D), together with left‐sided pleural effusion. Accordingly, he was tentatively diagnosed with a severe rapidly progressing biphasic malignant mesothelioma.

**Figure 2:**
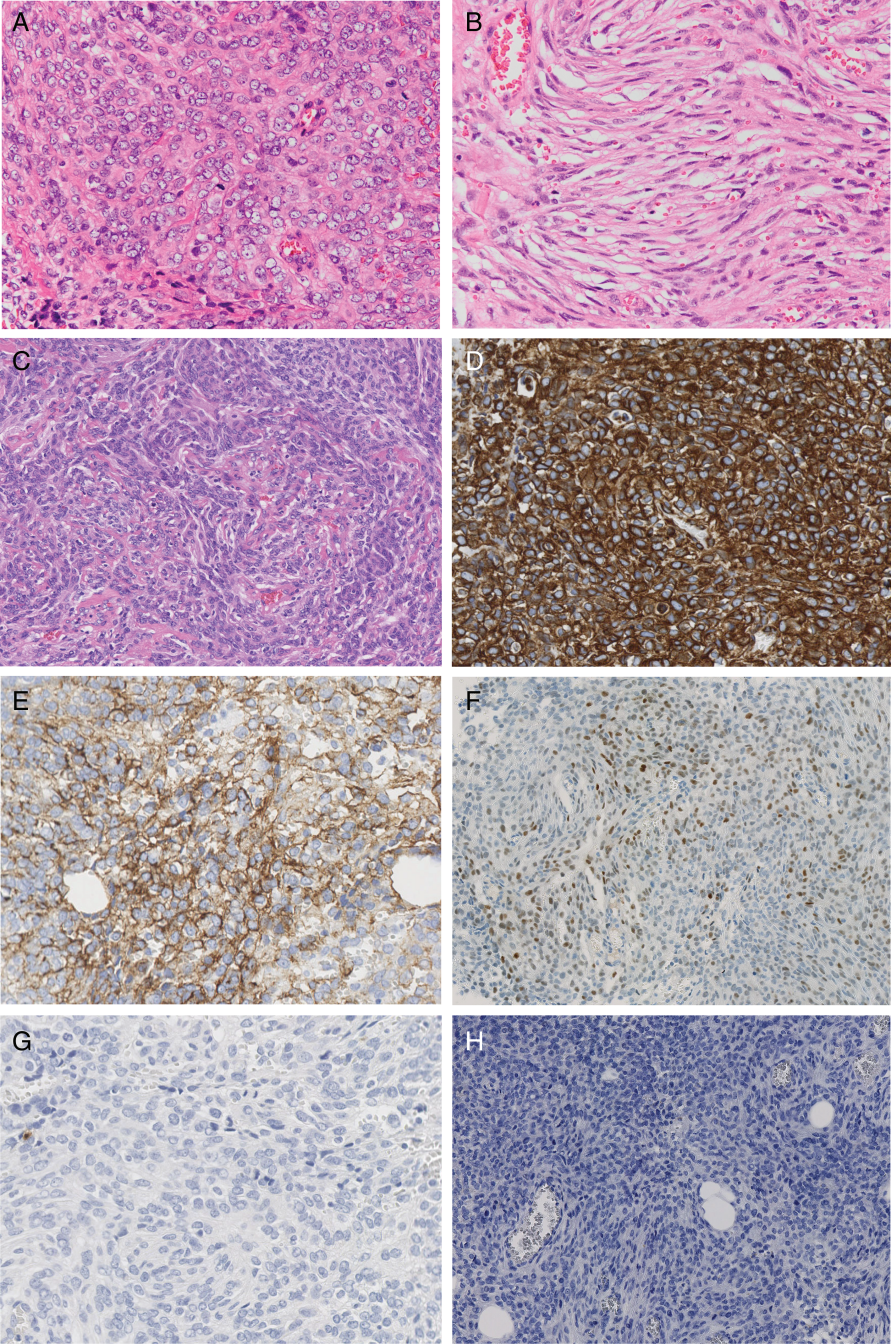
(A) Pleural samples showing an accumulation of abundant atypical epithelioid cells (H‐E* stain). (B) Pleural samples showing a large number of fusiform cells (H‐E stain). (C) Brain biopsy tissue showing a similar pattern (H‐E stain). (D) Atypical pleural cells positive for vimentin. (E) Atypical pleural cells positive for EMA. (F) Atypical pleural cells positive for progesterone receptor. (G) Atypical pleural cells negative for calretinin. (H) Atypical pleural cells negative for keratin CAM 5.2. H‐E, haematoxylin–eosin; EMA, epithelial membrane antigen. All slides were taken at 200 times magnification.

We re‐evaluated the pathological specimens retrieved from the local hospital. On haematoxylin–eosin (H‐E) staining, pleural samples showed abundant atypical epithelioid cells (Fig. 2A, 200×) as well as a large number of fusiform cells (Fig. 2B, 200×), indicating the possibility of meningioma (Fig. 2C, 200×) rather than malignant mesothelioma.

To confirm the pathological diagnosis, immunohistochemical staining was applied to the atypical pleural cells, which proved to be positive for vimentin (Fig. 2D, 200×), epithelial membrane antigen (EMA) (Fig. 2E, 200×), and progesterone receptor (Fig. 2F, 200×). Moreover, cytokeratin (CK) 5/6, WT‐1, and D2–40 were weakly positive, while results for calretinin (Fig. 2G, 200×), keratin CAM 5.2 (Fig. 2H, 200×), CK AE1/AE3, and oestrogen receptor were negative. In addition, Ki‐67‐positive cells accounted for over 50% of the atypical pleural cells.

Therefore, he was finally diagnosed with pleural metastasis secondary to anaplastic meningioma (WHO classification, grade 3). One month following the diagnosis, he died of respiratory failure.

## Discussion

The present case demonstrated that meningioma (also known as malignant meningioma) could metastasize to the pleura and that metastatic pleural meningioma seems to resemble malignant mesothelioma radiologically and pathologically.

With regard to all types of metastatic meningioma, Enam et al. 1 have reported that the incidence of metastasis to other organs was only 0.76%. The exact frequency of meningioma metastasizing to the pleura is unknown, but previous studies have reported the range to be from 5.5% 2 to 9.0% 4, suggesting that it is an extremely rare event. However, for WHO grade 3 meningioma 3, the rate of metastasis to other organs was as high as 42.8% 1, and only 4.3% to the pleura 2.

In terms of radiological findings, the present case seemed to resemble malignant mesothelioma; however, it was distinguished by the rapid expansion of the pleural thickening, which covered the entire right hemithorax in 1 month, and the absence of thoracic deformity, regardless of the severity of the pleural thickening. Furthermore, the negative results of the immunohistochemical staining for calretinin and cytokeratin in the atypical epithelioid cells excluded the possibility of identifying them as epithelial or biphasic malignant mesothelioma cells.

In conclusion, brain meningiomas, generally considered benign tumours, could rapidly and aggressively metastasize to the pleura as in the present case.

### Disclosure Statement

Appropriate written informed consent was obtained for publication of this case report and accompanying images.
